# Thalamic volume reduction in drug‐naive patients with new‐onset genetic generalized epilepsy

**DOI:** 10.1111/epi.13955

**Published:** 2017-11-18

**Authors:** Suejen Perani, Tim M. Tierney, Maria Centeno, Elhum A. Shamshiri, Siti N. Yaakub, Jonathan O'Muircheartaigh, David W. Carmichael, Mark P. Richardson

**Affiliations:** ^1^ Department of Basic and Clinical Neuroscience Institute of Psychiatry, Psychology and Neuroscience King's College London London United Kingdom; ^2^ Developmental Imaging and Biophysics Section Developmental Neurosciences Program UCL Great Ormond Street Institute of Child Health London United Kingdom; ^3^ Department of Neuroimaging Institute of Psychiatry, Psychology and Neuroscience King's College London London United Kingdom; ^4^ Centre for the Developing Brain Division of Imaging Sciences and Biomedical Engineering King's College London London United Kingdom

**Keywords:** drug naive, genetic generalized epilepsy, new onset, thalamus, volumetric MRI

## Abstract

**Objective:**

Patients with genetic generalized epilepsy (GGE) have subtle morphologic abnormalities of the brain revealed with magnetic resonance imaging (MRI), particularly in the thalamus. However, it is unclear whether morphologic abnormalities of the brain in GGE are a consequence of repeated seizures over the duration of the disease, or are a consequence of treatment with antiepileptic drugs (AEDs), or are independent of these factors. Therefore, we measured brain morphometry in a cohort of AED‐naive patients with GGE at disease onset. We hypothesize that drug‐naive patients at disease onset have gray matter changes compared to age‐matched healthy controls.

**Methods:**

We performed quantitative measures of gray matter volume in the thalamus, putamen, caudate, pallidum, hippocampus, precuneus, prefrontal cortex, precentral cortex, and cingulate in 29 AED‐naive patients with new‐onset GGE and compared them to 32 age‐matched healthy controls. We subsequently compared the shape of any brain structures found to differ in gray matter volume between the groups.

**Results:**

The thalamus was the only structure to show reduced gray matter volume in AED‐naive patients with new‐onset GGE compared to healthy controls. Shape analysis revealed that the thalamus showed deflation, which was not uniformly distributed, but particularly affected a circumferential strip involving anterior, superior, posterior, and inferior regions with sparing of medial and lateral regions.

**Significance:**

Structural abnormalities in the thalamus are present at the initial onset of GGE in AED‐naive patients, suggesting that thalamic structural abnormality is an intrinsic feature of GGE and not a consequence of AEDs or disease duration.

**Key Points**

- Brain morphology was studied in newly diagnosed AED‐naive patients with GGE for the first time
- Bilateral thalamus was smaller in patients than in healthy controls
- Thalamic volume loss in GGE is present at disease onset, independent of AEDs and repeated seizures
- We proposed that thalamic volume loss in GGE is a disease endophenotype

## 1 INTRODUCTION

People with genetic generalized epilepsy (GGE) are expected to have normal neuroimaging findings when studied with a conventional clinical magnetic resonance imaging (MRI) scan.^1^, ^2^ However, subtle abnormalities of brain morphology have frequently been reported. There are conflicting findings regarding which brain regions are involved and whether the abnormality consists of increased or reduced volume of particular brain regions. Gray matter atrophy in the putamen,^3^, ^4^ caudate,^4^ globus pallidus^3^ and hippocampus^5^ has been identified inconsistently across studies in patients with GGE compared to healthy controls. The thalamus has shown the most consistent differences, typically volume loss^3^, ^4^, ^6^, ^7^ in patients with GGE. Morphologic abnormalities of cortex, in particular prefrontal, precentral, and posterior cingulate, have also been reported, although inconsistently.^6^, ^8^, ^9^, ^10^, ^11^

Prior studies of brain morphology in GGE have been carried out in populations of patients with a longstanding diagnosis and on long‐term antiepileptic drug (AED) treatment; both factors could affect brain morphology.^12^, ^13^ This precludes unequivocal attribution of abnormalities in brain morphology to the underlying etiology. There is only one previous study that reported decreased thalamic volume in a group of drug‐naive patients with GGE, in this case children with childhood absence epilepsy (CAE), although they were studied on average a year after the first diagnosis.^14^

In the work reported herein, we measured brain morphology at the earliest possible time point in AED‐naive patients diagnosed with GGE. We hypothesize that AED‐naive patients at disease onset will have differences in gray matter volume compared to age‐matched healthy controls. All subjects were recruited through rapid‐access first seizure clinics and scanned within a week of initial diagnosis, before commencing AEDs.

## 2 METHODS

### 2.1 Subjects

We acquired data from 29 AED‐naive patients with GGE (mean age 15.07, standard deviation [SD] 7.8 years, range 5.9‐37.0 years) recruited from first seizure clinics across London. Inclusion criteria were the following: (1) diagnosis of GGE made by an epilepsy specialist following examination and investigation; (2) normal cognitive development; and (3) if a child, judged by their treating clinician to be capable of undergoing an MRI scan without sedation. Exclusion criteria were the following: (1) a history of any neurologic condition other than epilepsy; and (2) current use of drugs active in the brain (prescribed medications and drug or alcohol misuse).

We studied 32 age‐matched healthy controls (mean age 16.9, SD 7.3 years, range 6.6‐32.0 years) to compare with the AED‐naive patients with GGE (Mann‐Whitney *U *=* *383.5, *P *=* *.245). Inclusion criteria were if a child, judged to be capable of undergoing MRI scan without sedation. Exclusion criteria were the following: (1) current use of drugs active in the brain (prescribed medications and drug or alcohol misuse); (2) history of any neurologic or neurodevelopmental conditions; and (3) family history of epilepsy in any first‐degree relative. The study was approved by the Riverside Research Ethics Committee (REC approval number 12/LO/2006) and by the London‐Surrey Border Research Ethics Committee for the acquisition of pediatric healthy controls (REC approval number 11/LO/1421). Written informed consent was obtained from all participants. A parent or the nominated legal carer gave informed and written consent on behalf of the participant if younger than the age of 18.

A full list of the demographic characteristics of the patients is shown in Table 1.

**Table 1:** Demographics and clinical details of the patients

Gender	Age at scan (Y)	Syndrome	Seizures
M	6.0	CAE	Absence
M	10.5	JAE	Absence
M	6.3	CAE	Absence
M	10.5	JAE	Absence
F	13.6	JAE	Absence
M	8.0	CAE	Absence
F	7.7	CAE	Absence
M	10.2	JAE	Absence
F	9.7	CAE	Absence
M	7.1	CAE	Absence
M	9.2	CAE	Absence
M	5.9	CAE	Absence
M	6.8	JAE	Absence, GTCS
F	13.5	JAE	Absence
M	21.7	GTCSO	GTCS
M	16.7	GTCSO	GTCS
F	16.5	JME	Myoclonus, GTCS
F	13.6	JME	Myoclonus, GTCS
F	20.1	JME	Myoclonus, GTCS
F	15.3	JME	Myoclonus, GTCS
F	17.6	JME	Myoclonus, GTCS
F	26.0	JME	Myoclonus, GTCS
M	14.4	JME	Myoclonus, GTCS
F	25.9	GTCSO	GTCS
F	31.2	JME	Myoclonus, GTCS
F	16.4	GTCSO	GTCS
M	20.8	JME	Myoclonus, GTCS
F	19.1	JME	Myoclonus, GTCS
F	37.0	GTCSO	GTCS

F, female; M, male; CAE, childhood absence epilepsy; JAE, juvenile absence epilepsy; GTCSO, generalized tonic–clonic seizure only; JME, juvenile myoclonic epilepsy; GTCS, generalized tonic–clonic seizure; Y, age in years.

### 2.2 MRI acquisition

Fourteen patients and 16 healthy controls from age 5 to 13 were studied at UCL Great Ormond Street Institute of Child Health, London, using a three‐dimensional (3D) isotropic T1‐weighted FLASH sequence in the sagittal plane (echo time [TE] 4.94 msec, repetition time [TR] 11 msec, acquisition matrix 192 × 256 × 176, field of view 256 mm, voxel size 1 × 1 × 1 mm) using a 1.5T Siemens Avanto scanner. Fifteen patients and 16 healthy controls from age 13 to 37 were studied at King's College London using a 3D T1‐weighted IR‐SPGR sequence in the sagittal plane (TE 3.016 msec, TR 7.312 msec, acquisition matrix 256 × 256 × 196, field of view of 270 mm, voxel size 1.05 × 1.05 × 1.2 mm) using a 3T 750 General Electric scanner.

### 2.3 MRI analysis

Based on published literature in the field we hypothesized that the effects of interest would be seen in a set of candidate brain regions: bilateral thalamus, putamen, caudate, pallidum, hippocampus, precuneus, prefrontal cortex, precentral cortex, and cingulate. We therefore confined our analysis to these regions.

### 2.4 Volumetric analysis (subcortical and cortical)

**Figure 1:**
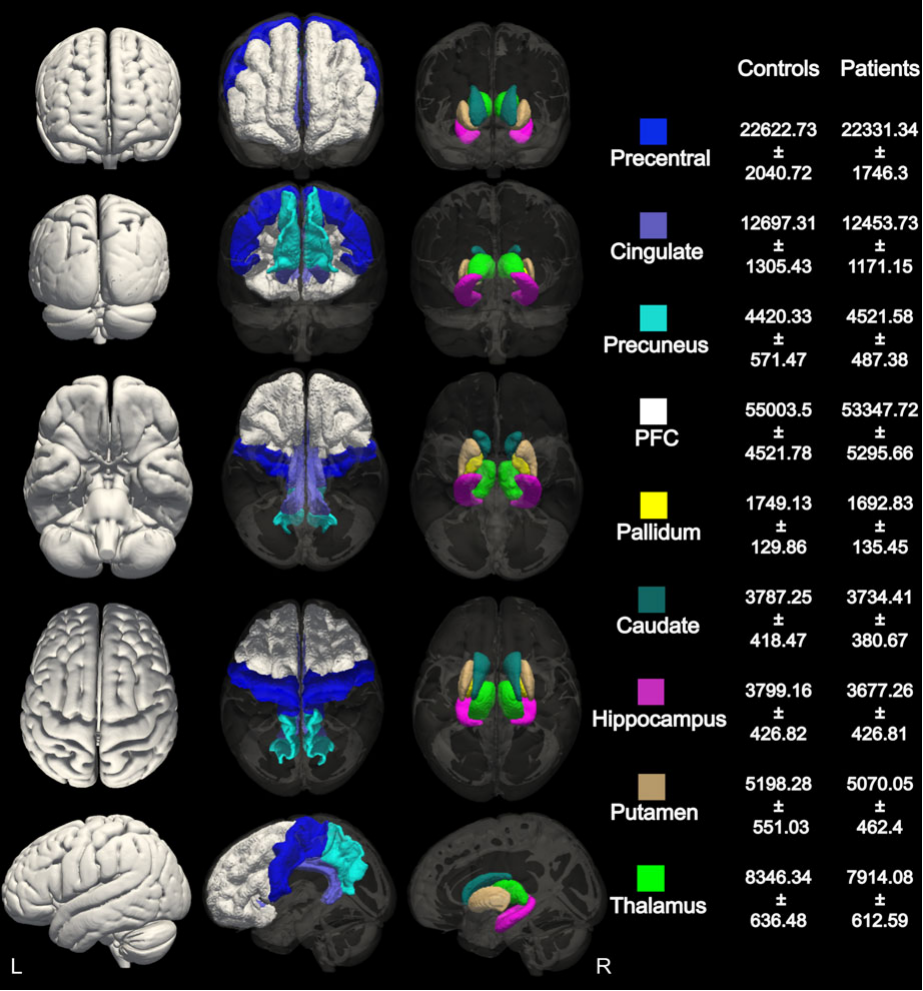
Cortical and subcortical volumetric segmentation. Gray matter masks of the cortical and subcortical areas selected for volumetric analysis between patients and controls. Average volumes and standard deviations for each area in the healthy control and AED‐naive group are reported in mm^3^

We used FSL‐integrated registration and segmentation toolbox (FSL‐FIRST) software, version 5.0 (http://fsl.fmrib.ox.ac.uk/fsl/fslwiki/first) (Analysis Group, FMRIB, Oxford, United KIngdom) for the automated segmentation of bilateral thalamus, putamen, caudate, pallidum, and hippocampus. As described in detail elsewhere,^7^, ^15^ FSL‐FIRST automatically segmented the subcortical structures from the MR images and produced volumetric outputs. Cortical regions of interest (ROIs) were segmented using standard “recon‐all” processing stream implemented in Freesurfer software (http://freesurfer.-net/)^16^ (The General Hospital Corporation, Boston, MA, USA) which supplies the surfaces and morphometry data for each subject. FSLstats (http://fsl.fmrib.ox.ac.uk/fsl/fslwiki/Fslutils) (Analysis Group, FMRIB) was used to measure each structure's global volume (Figure 1).

Left and right gray matter volumes were extracted separately, and the mean volume for each ROI was calculated as the mean across hemispheres. Normalized brain volume (NBV) was measured using SienaX^17^, ^18^ and included as a covariate in the analyses of cortical and subcortical areas to control for its effect. This methodologic choice was supported by evidence showing that volumes of the subcortical areas of interest are correlated positively with the cranium size^19^ and that SienaX measures are an excellent surrogate for the intracranial vault volume.^20^

### 2.5 Shape analysis

FSL‐FIRST outputs parameterized volumetric labels (meshes) from surfaces after affine registration of raw T1‐weighted images to Montreal Neurological Institute (MNI) 152 space. Vertex‐wise shape analysis along the surface of the segmented structures was performed via the measures of differences in mean vertex position between AED‐naive patients and healthy controls^7^, ^15^ only for those ROIs where differences had been found in the volumetric analysis using FSL software.

### 2.6 Statistical methods

Volumetric measures of cortical and subcortical areas were included in a general linear model as the dependent variables. For each area, a comparison was made between AED‐naive patients and controls, modeling age, gender, scanner type, and NBV as covariates. Note that because these covariates (age, gender, scanner type, and NBV) are effects of no interest, any correlations between them are unimportant. A Bootstrapping procedure was used to determine statistical significance so that parametric assumptions regarding model error were avoided. The data were sampled with replacement 5000 times. False discovery rate (FDR) was used to control for multiple comparisons across the 9 ROIs (*q *<* *0.05). Results are presented with regression coefficients (β) representing brain volume changes and 95% confidence interval (CI). For the shape analysis, 5000 permutation testing using “randomize”^21^ was used to calculate statistics from surface meshes of the segmented structures included in the general linear model (GLM), which were thresholded at *q *<* *0.05 and corrected for multiple comparisons using FDR. Age, gender, scanner type, and NBV were also included as covariates in this model.

## 3 RESULTS

Twenty‐nine patients and 32 controls were included in the analysis. The quality of subcortical and cortical segmentation was inspected visually. All patients passed a quality check for the subcortical segmentation in which the segmented areas were overlaid on each individual T1‐weighted image to verify anatomic correspondence between areas of interest. However, 3 patients were excluded from the cortical volumetric measures due to motion corruption that prevented accurate cortical segmentation.

### 3.1 Volumetric analysis

We found a reduction in the volume of the thalamus averaged across hemispheres in newly diagnosed AED‐naive patients compared to healthy controls, corrected for multiple comparisons using FDR (corrected *q *=* *0.032, β = 434.322, CI [179.162, 717.241]). Putamen (uncorrected *P *=* *.03, corrected *q *=* *0.1, β = 210.88, CI [12.687, 453.675]) and pallidum (uncorrected *P *=* *.01, corrected *q *=* *0.06, β = 74.6, CI [20.642, 134.852]) showed a trend toward volume reduction in newly diagnosed AED‐naive patients compared to healthy controls but did not reach statistical significance after correction for multiple comparisons.

Hippocampus, caudate, prefrontal cortex, precentral cortex, precuneus, and cingulate volumes did not show any differences between newly diagnosed AED‐naive patients compared to healthy controls (Figure 1).

Differences found in the subcortical volumes were not influenced by different acquisitions, as we found no statistically significant difference in the thalamic volume of the healthy controls between scanners (*F*[2,29] = 0.014, *P* = .880).

### 3.2 Shape analysis

**Figure 2:**
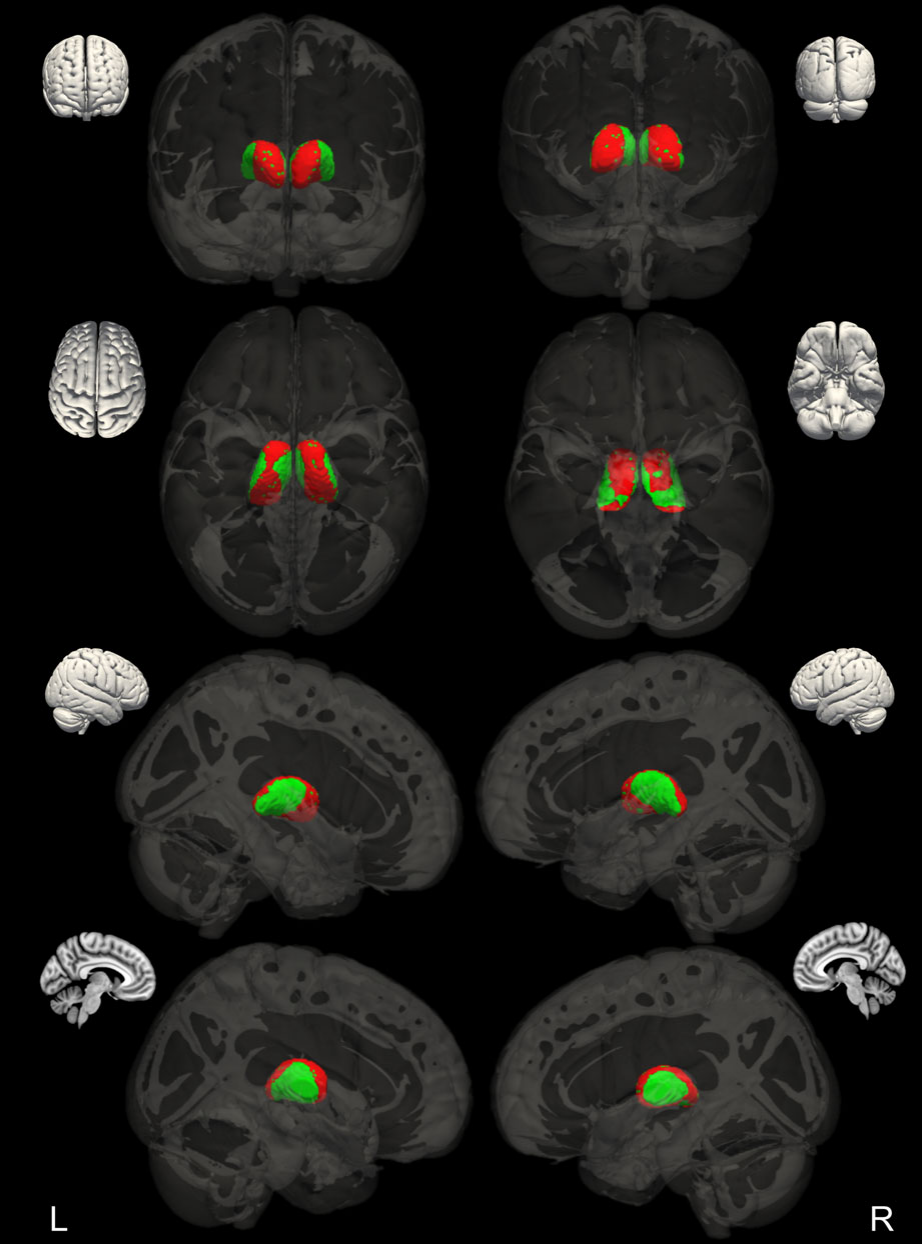
AED‐naive thalamic shape change. Bilateral changes in thalamic shape between healthy controls and AED‐naive patients are in red; the mask of thalamus is in green

**Figure 3:**
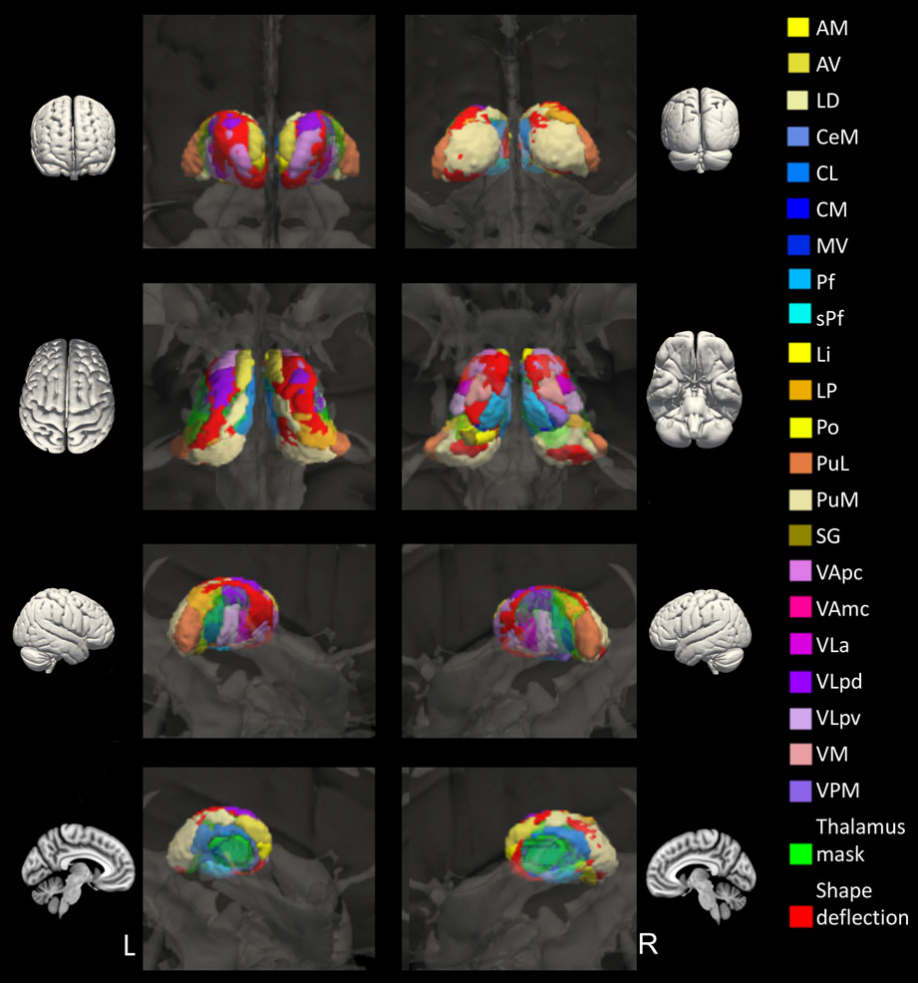
Correspondence between thalamic shape change and thalamic nuclei. Overlay of the Morel atlas on regions showing shape change comparing AED‐naive patients with GGE and healthy controls. A list of thalamic nuclei is on the right. Medial group: MV, medioventral nucleus; CL, central lateral nucleus; CeM, central medial nucleus; CM, centromedian nucleus; Pf, parafascicular nucleus; sPf, subparafascicular nucleus. Posterior group: PuM, medial pulvinar; PuL, lateral pulvinar; LP, lateral posterior nucleus; SG, suprageniculate nucleus; Li, limitans nucleus; Po, posterior nucleus. Lateral group: VPM, ventral posterior medial nucleus; VLa, ventral lateral anterior nucleus; VLpd/VLpv, ventral anterior nucleus dorsal and ventral parts; VAmc/VApc, ventral anterior nucleus magnocellular and parvocellular parts; VM, ventral medial nucleus. Anterior group: AM, anterior medial nucleus; AV, anterior ventral nucleus; LD, lateral dorsal nucleus

We explored the shape of bilateral thalami, as this was the only ROI showing significant volumetric difference between AED‐naive patients with new‐onset GGE and healthy controls. We found that left and right thalami showed differences in shape between the groups. There was relative deflation affecting a circumferential strip running in an anterior‐posterior orientation, incomplete inferiorly, with sparing of medial and lateral aspects (Figure 2). The results of the shape analysis are displayed in Figure 3, where they are overlaid on the Morel atlas.^22^

## 4 DISCUSSION

For the first time, we measured brain morphology in an AED‐naive population with GGE, within a week of first diagnosis, in which history suggested a seizure onset very close to the time of diagnosis. Our unique sample of patients allowed us to separate gray matter morphologic abnormalities from the effects of AED treatment and disease duration, which have confounded results of prior studies in this field. Our AED‐naive newly diagnosed patient group showed a decrease in volume of the thalamus compared to healthy controls, associated with deflation of the anterior, posterior, and midline aspects and relative sparing of medial and lateral aspects. In this study, we did not find evidence of morphologic abnormality in putamen, caudate, pallidum, hippocampus, precuneus, prefrontal cortex, precentral cortex, or cingulate.

Thalamus is the brain area that has shown most consistent differences between patients with established GGE on AED treatment and healthy control subjects across studies, typically revealing volume loss. The pattern of thalamic volume loss that has been reported to be atypical in GGE is consistent with our results. For example, patients with generalized tonic–clonic seizures only (GTCSO) revealed reduced gray matter in the ventral anterior and lateral nuclei of the thalamus compared to healthy controls.^3^, ^23^, ^24^ In a group of patients with juvenile myoclonic epilepsy (JME), shape analysis showed surface reduction in the medial and lateral nuclei of bilateral thalami^25^ and decreased gray matter volume in the anteromedial thalamus has also been found in patients with JME^11^ and in a group of mixed GGE patients.^26^ Similarly, gray matter volume reduction in bilateral thalami has been reported in patients with CAE.^9^ However, inconsistent results of increased thalamic gray matter volume was reported in CAE^27^; and no difference between GGE patients and healthy controls has also been reported.^4^

Although there is more consistent evidence supporting thalamic volume loss in GGE, additional areas of the basal ganglia have also been found abnormal in patients with GGE, although more inconsistently. For example, deflation of the putamen and pallidum was reported in a group of patients with GTCSO^3^; and in a further group with GTCSO, gray matter reduction was found in the cerebellum.^10^ In a group of patients with JME, shape analysis showed surface reduction in bilateral hippocampi.^25^ In an exploratory analysis of our data, we looked for shape differences between our GGE patients and healthy controls in hippocampi, putamen, and caudate; we found no significant shape differences in any part of any of these regions.

A similar inconsistency has been found in abnormalities of cortical areas. For example, an increase in volume has been found in the medial frontal cortex of patients with JME,^11^, ^28^ whereas another study of JME patients found a decrease in a similar region.^8^ In addition, medial frontal gyrus was found to have decreased gray matter volume in groups of patients with GTCSO.^10^, ^23^ In our study, we did not find any cortical areas of differences between GGE patients and healthy control, unlike these previous reports. Furthermore, our results did not show abnormalities in any subcortical areas in addition to the thalamus. However, given that a trend in decreased gray matter volume in putamen and pallidum was found, and in accordance with a previous report studying long‐term GGE patients on medication,^3^ we can speculate that changes in these structures may become more pronounced due to the potential effects of disease duration and medication.^29^

Whether different GGE syndromes have features or endophenotypes in common is a matter of debate; here, we have assumed thalamic atrophy may be a shared feature between GGE syndromes. Although it would have been of interest to explore potential differences between syndromes, we could not test for differences between syndromes independent from the effect of scanner and imaging sequence, although there is evidence showing that measurements made on different scanners are reliable.^19^, ^30^ This was due to the practical and ethical constraints of the study, where children were scanned on a 1.5T scanner at a pediatric focused site and adults on a 3T scanner at a different site.

All the previously published reports are derived from populations of patients entirely composed of, or biased toward, (1) refractory GGE, (2) taking long‐term AED treatment, and (3) with long disease durations. These confounding factors may account for the contrasting results reported between groups of patients and groups of healthy controls across studies. For example, gray matter volume (cortical and subcortical) has been reported to be negatively correlated with disease duration in GGE,^7^, ^10^, ^14^, ^24^ although some studies did not find thalamic volume and disease duration to be correlated,^3^, ^23^ which may relate to the patient group selected, or to other factors such as seizure frequency or AED treatment. The effect of AED treatment is unclear: although reports of animal studies have indicated effects on brain development, there are only case reports available in humans that confirm these effects.^13^, ^31^, ^32^

In our study, we investigated drug‐naive patients with GGE at the immediate onset of their condition, within a few days of first presentation to a rapid‐access first seizure clinic. Selecting only drug‐naive GGE patients at the immediate onset of their condition has allowed us to control for disease duration and medication effects. There is only one previous study that reported decreased gray matter volume in a group of drug‐naive CAE early in their disease.^14^ However, these patients were studied at an average of 1.86 ± 0.86 years between disease onset and time of scanning, which is potentially significant in the context of interactions between ongoing brain growth and development and seizures. Hence it is not clear whether the thalamic morphologic abnormality reported would have been present at the onset of epilepsy. Furthermore, in a cohort of CAE patients scanned twice (baseline within a few months of first diagnosis and then again 2 years later) and who were receiving AED treatment at both time points, there was evidence of thalamic volume loss at the end of the 2‐year interval, suggesting that disease and/or treatment factors may impact thalamic volume early in the disease course.^33^ It is important to note that evidence from our study suggests that thalamic volume loss is present at epilepsy onset and is therefore likely to be an underlying disease feature, not a consequence of AED treatment or recurring seizures over a prolonged period.

The involvement of the thalamus in the pathogenesis of generalized spike‐wave discharges (GSWs), the electroencephalography (EEG) hallmark of GGE, has been well established. Thalamocortical circuits are believed to play an important role in the pathogenesis of GGE in experimental animal models.^34^ In humans, electrophysiologic studies demonstrate that the thalamus is integral to seizure generation^35^; EEG–functional MRI (fMRI) studies have shown thalamic activation and cortical deactivation during GSW^36^, ^37^; and brain connectivity studies indicate thalamocortical circuit abnormalities in patients with GGE.^38^ However, the pathophysiologic mechanisms explaining the relationship between GGE and thalamic volume loss are still unknown. One possible mechanism might relate to genetic variation in bromodomain containing 2, reported in JME patients^39^ which has been found to lead to γ‐aminobutyric acid (GABA)ergic neuron deficit found in mice^40^; this might associate with thalamic volume loss as reported here.

Although our approach did not allow us to identify individual thalamic nuclei, abnormalities of thalamic shape appeared to be in the vicinity of the pulvinar, anterior nuclei, lateral posterior, and lateral dorsal nuclei. These areas connect to cingulate, caudate, putamen, frontal cortex, supplementary motor area, postcentral cortex, and occipital cortex,^41^ all cortical areas that are associated with GGE.^36^, ^42^, ^43^

For the first time, we have demonstrated that thalamic volume loss is present at the onset of GGE in AED‐naive patients. From this evidence, we suggest that thalamic volume loss is related to disease mechanisms and not solely a consequence of treatment or longstanding seizures.

## DISCLOSURE

None of the authors has any conflict of interest to disclose. We confirm that we have read the Journal's position on issues involved in ethical publication and affirm that this report is consistent with those guidelines.
